# Regulation of Myosin Light Chain Kinase during Insulin-Stimulated Glucose Uptake in 3T3-L1 Adipocytes

**DOI:** 10.1371/journal.pone.0077248

**Published:** 2013-10-08

**Authors:** Shelly Woody, Richard Stall, Joseph Ramos, Yashomati M. Patel

**Affiliations:** Department of Biology, University of North Carolina at Greensboro, Greensboro, North Carolina, United States of America; University of Chicago, United States of America

## Abstract

Myosin II (MyoII) is required for insulin-responsive glucose transporter 4 (GLUT4)-mediated glucose uptake in 3T3-L1 adipocytes. Our previous studies have shown that insulin signaling stimulates phosphorylation of the regulatory light chain (RLC) of MyoIIA via myosin light chain kinase (MLCK). The experiments described here delineate upstream regulators of MLCK during insulin-stimulated glucose uptake. Since 3T3-L1 adipocytes express two MyoII isoforms, we wanted to determine which isoform was required for insulin-stimulated glucose uptake. Using a siRNA approach, we demonstrate that a 60% decrease in MyoIIA protein expression resulted in a 40% inhibition of insulin-stimulated glucose uptake. We also show that insulin signaling stimulates the phosphorylation of MLCK. We further show that MLCK can be activated by calcium as well as signaling pathways. We demonstrate that adipocytes treated with the calcium chelating agent, 1,2-b (iso-aminophenoxy) ethane-N,N,N',N'-tetra acetic acid, (BAPTA) (in the presence of insulin) impaired the insulin-induced phosphorylation of MLCK by 52% and the RLC of MyoIIA by 45% as well as impairing the recruitment of MyoIIA to the plasma membrane when compared to cells treated with insulin alone. We further show that the calcium ionophore, A23187 alone stimulated the phosphorylation of MLCK and the RLC associated with MyoIIA to the same extent as insulin. To identify signaling pathways that might regulate MLCK, we examined ERK and CaMKII. Inhibition of ERK2 impaired phosphorylation of MLCK and insulin-stimulated glucose uptake. In contrast, while inhibition of CaMKII did inhibit phosphorylation of the RLC associated with MyoIIA, inhibition of CAMKIIδ did not impair MLCK phosphorylation or translocation to the plasma membrane or glucose uptake. Collectively, our results are the first to delineate a role for calcium and ERK in the activation of MLCK and thus MyoIIA during insulin-stimulated glucose uptake in 3T3-L1 adipocytes.

## Introduction

A critical component of whole body glucose homeostasis is insulin-stimulated glucose uptake into adipose tissue and skeletal muscle [1]. Insulin stimulates glucose uptake by inducing the translocation, docking and fusion of the insulin responsive glucose transporter 4 (GLUT4) to the plasma membrane. Insulin-stimulated glucose uptake requires the activation of several signaling pathways to mediate the trafficking of GLUT4 vesicles from an intracellular pool to their fusion with the plasma membrane [2]. The binding of insulin to its receptor activates the phosphatidylinositol-3 kinase (PI3K), mitogen activated protein kinase (MAPK), Cbl and Ca^2+^ signaling pathways [3]–[6]. All of these pathways are required for GLUT4 trafficking and glucose uptake in adipocytes.

While insulin does not cause dramatic changes in intracellular Ca^2+^ levels, Ca^2+^ is required for insulin-stimulated glucose uptake. Previous studies have shown that Ca^2+^ plays a role in two steps in insulin-stimulated glucose uptake [5], [6]. Chelating intracellular Ca^2+^ results in impaired GLUT4 vesicle translocation and fusion with the plasma membrane [5], [6]. Insight into a potential mechanism of action of Ca^2+^ in GLUT4 vesicle trafficking comes from the known function of Ca^2+^ in other exocytic processes (reviewed in [7]). Ca^2+^ has been shown to be required for GLUT4 vesicle fusion with the plasma membrane [8]. Vesicle fusion requires actin reorganization and the regulation of other cytoskeletal structures at the cell cortex. While filamentous actin (F-actin) reorganization has also been implicated in GLUT4 trafficking and insulin-stimulated glucose uptake [9] little is known about the contractile activities/forces involved in regulating actin reorganization.

Mature adipocytes do not have an extensive array of stress fibers but instead have a layer of cortical actin filaments that line the inner surface of the plasma membrane [10]. Previous studies have demonstrated that insulin-stimulated GLUT4 translocation and membrane fusion in adipocytes requires cortical actin reorganization [9]. In adipocytes the actin cytoskeleton functions as a barrier (or shell) at the cell cortex which must be “loosened/relaxed” in order for vesicles to fuse with the plasma membrane. To accomplish this function the actin cytoskeleton requires the myosin family of actin-based motor proteins. Members of the myosin family have been shown to contract actin filaments [11]. Contraction of the acto-myosin cytoskeleton can lead to the localized membrane remodeling required for vesicle fusion at the plasma membrane.

The myosin responsible for actin filament contraction is ‘conventional’ myosin, myosin II, (MyoII) [11]. Nonmuscle cells express MyoII isoforms that function in a manner similar to their muscle counterpart. In contrast to skeletal muscle MyoII, which is organized in a highly regular and stable arrangement with actin filaments in sarcomeres, nonmuscle MyoII can undergo dramatic changes in localization and activation as a part of various cellular events (reviewed in [12]). Previous studies have implicated a role for nonmuscle MyoII in vesicle transport and fusion [13]. These studies suggest that there are distinct zones at the cell cortex where myosin-dependent cytoskeletal reorganization occurs and allows for the localized membrane remodeling required for vesicle fusion with the plasma membrane. Previous studies have shown that MyoII plays a similar role in the GLUT4 vesicle trafficking required for insulin-stimulated glucose uptake [14]–[17].

Nonmuscle MyoII is a hexameric protein consisting of two heavy chains (MHC), two regulatory light chains (RLC) and two essential light chains (ELC) [11]. MyoII motor activity and parallel filament assembly are regulated by the phosphorylation of residues localized on the RLCs [11]. Phosphorylation of the RLC via myosin light chain kinase (MLCK) initiates the binding of MyoII to filamentous actin [18]–[20]. Our previous studies show that insulin specifically stimulates the phosphorylation of the RLC associated with the MyoIIA isoform via MLCK but not by RhoK [14]. In time course experiments, we determined that GLUT4 translocates to the plasma membrane independently of prior to MyoIIA recruitment [16]. We further show that recruitment of MyoIIA to the plasma membrane requires that MyoIIA be activated via phosphorylation of the RLC by MLCK [16], [17]. While MyoII activity is not required for GLUT4 translocation it is required for proper GLUT4-vesicle fusion at the plasma membrane [16]. What is not known is the mechanism by which MLCK is regulated during insulin-stimulated glucose uptake. MLCK can be activated by both a Ca^+2^-dependent and -independent manner. Increased levels of intracellular Ca^+2^ coupled with calmodulin activate MLCK resulting in the phosphorylation of the RLC of MyoII [21]. MLCK can also be activated by the MAPK (RAS/Raf/MEK/ERK) pathway in a Ca^+2^- independent manner [22], [23]. Both ERK and CaMKII have been shown to regulate MLCK phosphorylation in a variety of cellular processes [22]–[24].

In the present study we examined the mechanism by which insulin signaling mediates MLCK phosphorylation and thus MyoIIA and GLUT4-mediated glucose uptake in adipocytes. Our results reveal that insulin signaling via elevated levels of Ca^+2^ and the MAPK pathway activate MLCK via phosphorylation during insulin-stimulated glucose uptake. Thus our studies are the first to identify the insulin induced regulators of MLCK required for activation of MyoIIA and insulin-stimulated glucose uptake in adipocytes.

## Materials and Methods

### Materials

Tissue culture reagents were purchased from Gibco (Grand Island, NY). Insulin was obtained from Roche Diagnostics Corporation (Indianapolis, IN). Dexamethasone, 3-isobytyl-1-methyl-xanthine, 1,2-Bis (2-aminophenoxy) ethane-N,N,N′,N′-tetraacetic acid tetrakis (acetoxymethyl ester) (BAPTA) and myosin IIA antibody were purchased from Sigma (St. Louis, MO). A-23187 and 1-[N,O-*bis*-(5-Isoquinolinesulfonyl)-N-methyl-L-tyrosyl]-4-phenylpiperazine (KN-62) were purchased from Calbiochem (San Diego, CA). U-0126 was from Promega (Madison, WI). Myosin light chain (phosphor S20) antibody was purchased from Abcam (Cambridge, MA). GLUT4 antibodies (C-20 and N-20), MLCK antibody and protein A/G plus agarose beads were obtained from Santa Cruz Biotechnology (Santa Cruz, CA). Phospho-MLCK antibody was from Invitrogen (Carlsbad, CA). The myosin IIA and IIB antibodies were obtained from Covance (Berkeley, CA). Actin, phospho-myosin light chain (Thr 18/Ser 19) antibody, ERK1/2 and pERK1/2 antibodies were obtained from Cell Signaling (Danvers, MA). CaMKII delta antibody was a generous gift from Dr. Harold Singer (Albany Medical College, NY). Alexa Fluor® 594 donkey anti-goat IgG and Alexa Fluor 488 goat anti-rabbit IgG were from Molecular Probes, Inc. (Eugene, OR). The enhanced chemiluminescence (ECL) detection kit and horseradish peroxidase conjugated secondary antibodies were obtained from Amersham Bioscience (Piscataway, NJ).

### Cell Culture

3T3-L1 fibroblasts were obtained from the ATCC (Manassas, VA). Cells were grown to confluency and then induced to differentiate in 10% Fetal Bovine Serum (FBS)/Dulbecco's Modified Eagle Media (DMEM) with the addition of 0.52 mM 3-isobutyl 1-methyl-xanthine (MIX), 1.7 µM insulin, and 1 µM dexamethasone (DEX) on Day 0. On Day 2, the media was changed to 10%FBS/DMEM plus 0.425 µM insulin. The media was replaced with DMEM containing 10% FBS every two days [25], [26].

### siRNA Duplexes

The siRNA (Dharmacon, Inc., Lafayette, CO) for myosin IIA, IIB, MLCK, CaMKIIδ, ERK2 or a scrambled sequence was ON-TARGET plus Smart POOL.

### Electroporation

Day 8 3T3-L1 adipocytes were washed with a 1X PBS and solubilized using 0.5% trypsin-EDTA. After a 6 min incubation at 37°C, cells were resuspended in 10% FBS/DMEM and centrifuged for 5 min at 100×g. The supernatant was removed and the cells were resuspended in 1× PBS and centrifuged for 5 min at 100×g twice. The cell pellet was resuspended in 1 ml 1×PBS per 15 cm plate used. Cuvettes (4 mm gap) containing 1 nmol of the siRNA (water, Myosin IIA, Myosin IIB, Scramble, MLCK, CaMKIIδ, or ERK2) were placed on ice. Cells (2.5×10^6^) along with the 1 nmol of siRNA was electroporated at 0.18 kV and 975 microfarads using a Bio-Rad Gene Pulser II. Immediately after electroporation, cells were re-suspended in 8.5 ml of 10%FBS/DMEM. After a 10 minute incubation period at 25°C, cells were re-plated onto multi-well plates. After 24 h, media was replaced with 10% FBS/DMEM [27]–[29].

### Glucose uptake assay

3T3-L1 preadipocytes were induced to differentiate as described above. Glucose uptake assays were performed on fully mature 3T3-L1 adipocytes. Adipocytes were serum-starved for 4 h in the presence of 0.1% DMSO (Basal) or inhibitor as indicated. Adipocytes were washed twice with 37°C Krebs Ringer Phosphate (KRP) buffer (pH 7.4) containing 128 mM NaCl, 4.7 mM KCl, 1.65 mM CaCl_2_, 2.5 mM MgSO_4_, 5 mM Na_2_HPO_4_ and then placed in KRP buffer containing vehicle, 50 µM BAPTA-AM, 0.1 µM A23187, 25 µM U0126 or 100 µM KN-62 as indicated. Adipocytes were then either untreated (basal) or treated with insulin (100 nM) for 10 min, followed by [1-^14^C]-2-deoxy-D-glucose (0.1 µCi/well) (NEN) and 5 mM glucose for an additional 10 min at 37°C. Cells were washed three times with phosphate buffered saline (PBS) and solubilized in 0.5 M NaOH and 0.1% SDS. Samples were assayed for ^14^C-2-deoxy-D-glucose uptake as disintegrations per min per mg protein. Data are expressed as means ± SEM.

### Immunoblot analysis

Cells were lysed in a buffer containing 25 mM HEPES pH 7.4, 1% Nonidet P-40, 100 mM NaCl, 2% glycerol, 5 mM NaF, 1 mM EDTA, 1 mM Na_3_VO_4_, 1 mM NaPP_i_, 1 mM phenylmethylsulfonyl fluoride (PMSF), 10 mg/ml aprotinin, 5 mg/ml leupeptin, and 5 mg/ml pepstatin [9]. Cell lysates were incubated at 4°C for 20 min and then centrifuged at 6,000×g for 20 min at 4°C. Supernatants (100 µg) were incubated for 5 minutes at 95°C in Laemmli sample buffer [30], and then subjected to SDS-PAGE. Proteins were transferred to Immobilon-P membranes (Millipore), and analyzed by immunoblotting as previously described [31]. Protein bands were quantified by densitometry using ImageQuant (version 5.2 for Windows) software.

### Immunoprecipitation

Whole cell lysates (1 mg) were incubated with antibodies (3 µg/ml) overnight at 4°C. Protein A/G PLUS–agarose beads were added to the immunoprecipitates and incubated for 1 h at 4°C. Samples were subjected to centrifugation at 2500×g and then washed three times with ice cold lysis buffer. Immunoprecipitated proteins were dissolved in 5× Laemmli buffer and then subjected to SDS-PAGE and immunoblot analysis.

### Immunofluorescence

Adipocytes grown on coverslips were serum starved for 4 h and treated according to the glucose uptake protocol (omitting ^14^C-2-DOG). Cells were fixed with 3% buffered paraformaldehyde, and then permeablized in 0.25% triton X-100 for 5 min at 4°C. Cells were incubated with anti-GLUT4 antibody or anti-myosin IIA antibody. The cells were then incubated with the appropriate secondary labeled antibodies. Slides were viewed using an Olympus IX81 Motorized Inverted Confocal Microscope and FLUOVIEW FV5OO software. The relative intensity of immunofluroescence was quantified using Image-Pro Plus software (Silver Spring, MD).

### Statistical analysis

Results are expressed as mean ± SEM. Statistical significance was determined using unpaired Student's t test, with P<0.05 considered significant.

## Results

### Inhibition of MyoIIA alone impairs insulin-stimulated glucose uptake

**Figure 1:**
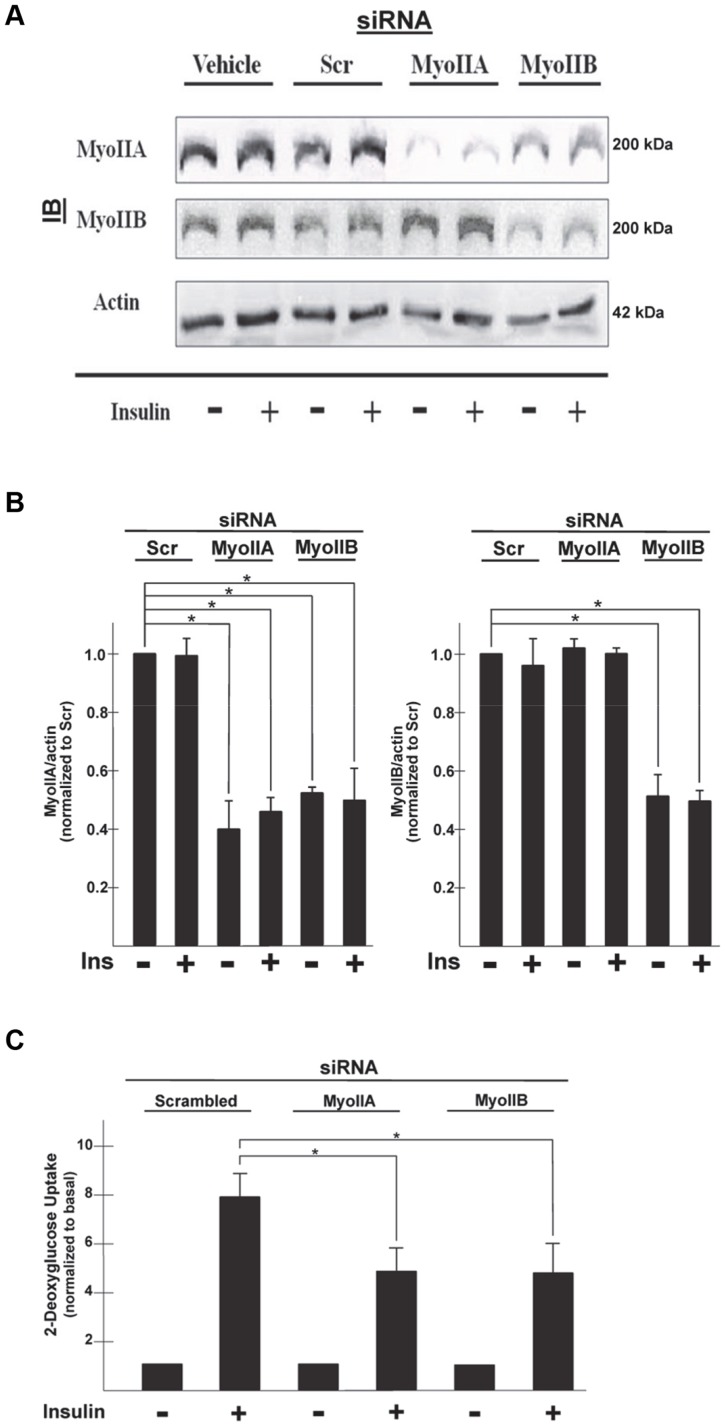
Knockdown of Myosin IIA impairs insulin-stimulated glucose uptake. 3T3-L1 adipocytes were either electroporated with vehicle (water) or with 1 nmole of siRNA (Scramble, MyoIIA, or MyoIIB). After 72 h cells were serum starved for 4 h and then either left untreated or stimulated with insulin (100 nM) for 20 min. (A) Whole cell lysates were prepared, subjected to SDS-PAGE and then immunoblotted (IB) using antibodies against myosin IIA, myosin IIB or actin. (B) Determination of band densities was measured using ImageQuant software and are presented as either MyoIIA/actin or MyoIIB/actin and normalized to basal levels in the Scr treated cells. Results are the mean ± SEM of three independent experiments. *p<0.05. (C) Untreated (Basal) or insulin stimulated (100 nM) adipocytes were incubated with [1-^14^C]-2-deoxy-D-glucose (0.1 µCi/well) and 5 mM glucose for 10 min. Cells were then lysed and glucose uptake calculated as disintegrations per mg protein and expressed as percent of the scrambled siRNA control. Results are the mean ± SEM of three independent experiments. *p<0.05.

Our previous studies using pharmacological inhibitors demonstrated that inhibition of myosin II resulted in impaired insulin-stimulated glucose uptake in 3T3-L1 adipocytes [16]. Since 3T3-L1 adipocytes express both myosin II isoforms, IIA and IIB, it is important to determine the role of each of the isoforms during insulin-stimulated glucose uptake. Since pharmacological inhibitors are not specific, we wanted to use a siRNA approach to determine which MyoII isoform was involved in insulin-stimulated glucose uptake. Fully differentiated adipocytes were electroporated with either vehicle or with siRNA (Scramble, MyoIIA, or MyoIIB). After 72 h cells were serum starved for 4 h and then either left untreated or stimulated with 100 nM insulin for 20 min. Western blots were performed to determine knockdown of the target protein in relation to actin. Cells electroporated with either vehicle or scrambled sequence had similar levels of MyoIIA and MyoIIB (Fig. 1A). In contrast, adipocytes electroporated with siRNA targeting MyoIIA had a 60% reduction in MyoIIA protein expression (Fig. 1A and B) and had no effect on MyoIIB protein levels when compared to vehicle-treated or scrambled siRNA electroporated cells. However, adipocytes electroporated with siRNA targeting MyoIIB had a 50% reduction in MyoIIB protein levels but also knocked down the expression of the myosin IIA by 50% (Fig. 1A and B).

Our previous studies have shown that the myosin IIA isoform is involved in insulin-stimulated glucose uptake [14], [16]. In order to determine the effect of knockdown of the myosin II isoforms on glucose transport we performed glucose uptake assays. Adipocytes electroporated with siRNA (Scramble, MyoIIA, or MyoIIB) were serum starved for 4 h and then either left untreated (−) or stimulated with 100 nM insulin (+) for 20 min in the presence of [1-^14^C]-2-deoxy-D-glucose and glucose uptake assays were performed. There was no effect on glucose uptake under basal conditions with the knockdown of either myosin IIA or IIB compared to cells electroporated with the scramble sequence. Insulin typically stimulates glucose uptake approximately five to ten-fold over basal levels. In adipocytes electroporated with a scrambled sequence, insulin stimulated an eight-fold increase in glucose uptake over basal levels (Fig. 1C). Insulin-stimulated glucose uptake was decreased by approximately 40% (7.9±0.90 versus 4.9±0.89, p<0.05) for MyoIIA knockdown cells as compared to cells electroporated with the scramble siRNA. There was also approximately a 40% decrease (7.9±0.90 versus 4.8±1.20, p<0.05) in insulin-stimulated glucose uptake in cells electroporated with MyoIIB siRNA when compared to cells electroporated with the scramble siRNA, however as observed from the western blot analysis, both MyoIIB and MyoIIA protein levels were knocked down in these cells making these results less conclusive. These studies suggest that knockdown of MyoIIA results in impaired insulin-stimulated glucose uptake and that knockdown of both MyoIIA and MyoIIB did not further impair glucose uptake. Thus, these finding provide further support that myosin IIA activity is required for insulin-stimulated glucose uptake.

### Insulin stimulates the phosphorylation of MLCK in 3T3-L1 adipocytes

**Figure 2:**
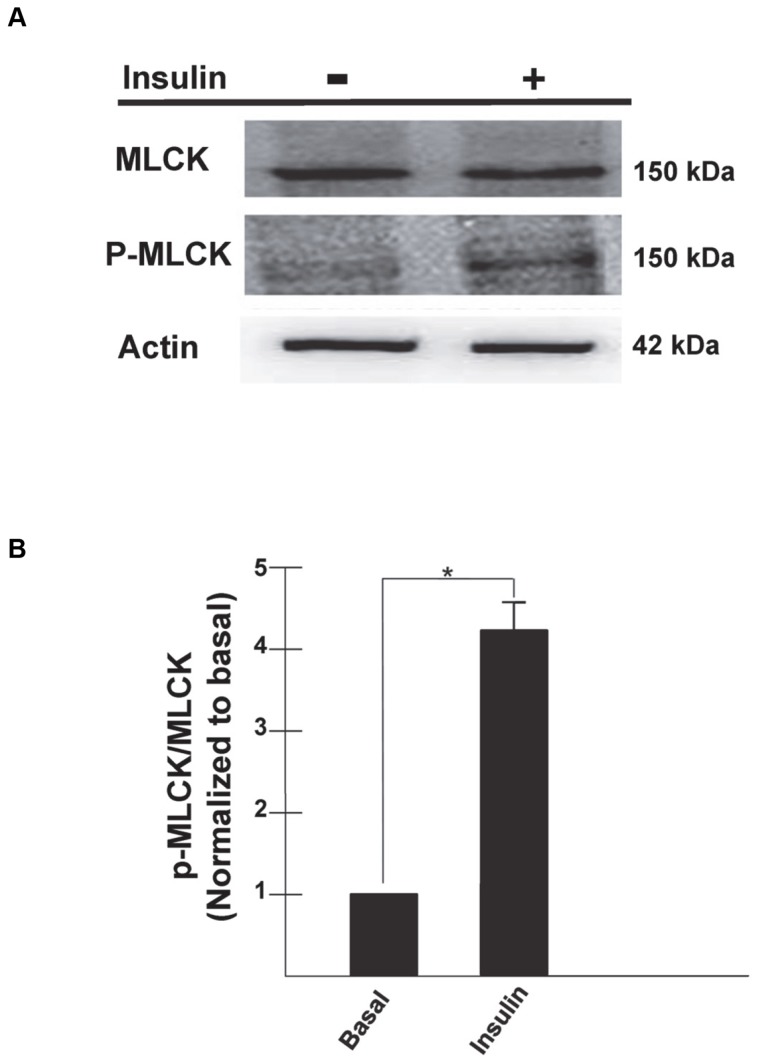
Insulin stimulates the phosphorylation of MLCK in 3T3-L1 adipocytes. 3T3-L1 adipocytes were serum starved for 4 h and then either left untreated or stimulated with insulin (100 nM) for 20 min. (A) Whole cell lysates were prepared, subjected to SDS-PAGE and then immunoblotted using antibodies against MLCK, P-MLCK, or actin. (B) Determination of band densities was measured using ImageQuant software and are presented as P-MLCK/MLCK and normalized to basal levels. Results are the mean ± SEM of three independent experiments. *p<0.05.

MyoII activity is regulated by the phosphorylation of residues localized on the RLCs [11], [18]–[20]. Our previous studies have shown that insulin specifically stimulates the phosphorylation of the RLC associated with the MyoIIA isoform via MLCK [16]. Inhibition of MLCK results in decreased phosphorylation of the RLC associated with MyoIIA and impaired insulin-stimulated glucose uptake [16]. In order to determine the mechanism by which the insulin signaling pathway regulates MLCK, we examined the phosphorylation status of the MLCK upon insulin stimulation. Previous studies have shown that MLCK activity is regulated by phosphorylation at Ser1760 [32]–[34]. Using a phospho-MLCK (Ser1760) specific antibody we observed that in the basal state only a low level of phospho-MLCK (p-MLCK) was detected in whole cell lysates (Fig. 2A). Upon insulin stimulation, there was a 4-fold increase in the level of p-MLCK (Fig. 2B). Our results also showed that the differences in the levels of p-MLCK were due to the degree of phosphorylation and not the levels of total MLCK since the absolute levels of MLCK were unchanged under all conditions (Fig. 2).

### Calcium regulation of MLCK facilitates GLUT4-mediated glucose uptake in 3T3-L1 adipocytes

In order to further delineate the signaling pathway(s) regulating MLCK during insulin-stimulated glucose uptake, we examined the role of calcium since previous studies have shown that MLCK is a Ca^2+^-regulated kinase and that inhibition of Ca^2+^ signaling impairs insulin-stimulated glucose uptake [21]–[24]. We wanted to determine whether insulin regulates MLCK in a Ca^2+^-dependent manner and reciprocally, whether Ca^2+^ can activate MLCK independently of insulin stimulation, so we employed the calcium chelator BAPTA-AM and the calcium ionophore A23187. Previous studies have shown that chelation of intracellular Ca^2+^, using BAPTA-AM significantly impaired insulin-stimulated glucose in 3T3-L1 adipocytes [5], [6]. In order to determine whether Ca^2+^ signaling regulates MLCK, mature 3T3-L1 adipocytes were subjected to either 50 µM BAPTA-AM or 0.1 µM A23187 for 20 min and then either left untreated or stimulated with insulin (100 nM) for an additional 20 min and assayed for glucose uptake, phosphorylation of MLCK, MyoIIA activation via phosphorylation of the RLC and MyoIIA localization as well as GLUT4 vesicle trafficking.

**Figure 3:**
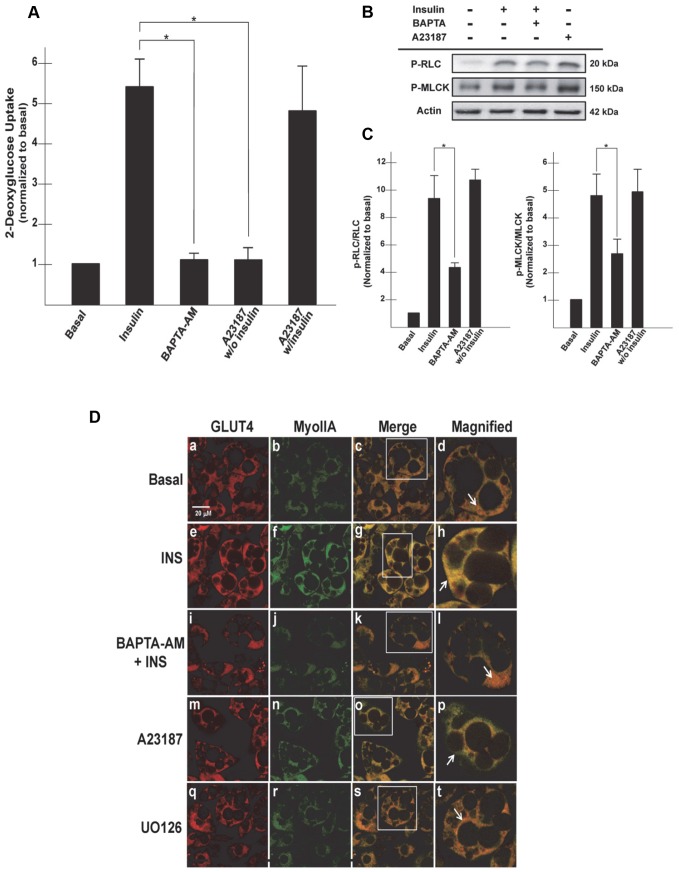
Calcium regulation of MLCK facilitates GLUT4-mediated glucose uptake in 3T3-L1 adipocytes. 3T3-L1 adipocytes were serum starved for 4 h then pretreated with BAPTA-AM (50 µM), A23187 (0.1 µM) or U0126 (25 µM) for 20 min and then either left untreated or stimulated with insulin (100 nM) for an additional 20 min. (A) Cells were untreated (Basal), treated with A23187 alone or insulin stimulated (100 nM) in the presence of A23187 and then incubated with [1-^14^C]-2-deoxy-D-glucose (0.1 µCi/well) and 5 mM glucose for an additional 10 min. Cells were then lysed and glucose uptake calculated as disintegrations per mg protein and expressed as percent of control. Results are the mean ± SEM of three independent experiments. Differences between insulin-stimulated cells and treated cells (BAPTA-AM or A23187) were tested for statistical significance (*p<0.05). (B) Whole cell lysates were prepared, subjected to SDS-PAGE and then immunoblotted using antibodies against either P-RLC or P-MLCK. (C) Determination of band densities was measured using ImageQuant software and are presented as either P-RLC/RLC or P-MLCK/MLCK and normalized to basal levels. Results are the mean ± SEM of three independent experiments. Insulin treated cells were compared to BAPTA-AM treated cells, *p<0.05. (D) Localization of GLUT4 and MyoIIA were examined by confocal microscopy (as described in Materials and Methods). The results are representative images from three independent experiments. The white arrows indicate either cytoplasmic or plasma membrane localization.

As shown previously, insulin stimulated approximately a five-fold increase in glucose uptake over basal levels (Fig. 3A). BAPTA-AM treatment completely prevented insulin-stimulated glucose uptake (Fig. 3A, INS versus BAPTA-AM, 5.4±0.61 versus 1.1±0.15, p<0.05). Glucose uptake was decreased to basal levels in adipocytes treated with BAPTA-AM in the presence of insulin. BAPTA-AM treatment also decreased phosphorylation of MLCK as well as the RLC associated with MyoIIA (Fig. 3B and C). Since our previous studies demonstrated that phosphorylation of the RLC of MyoIIA is required for MyoIIA translocation to the plasma membrane, we examined the localization of MyoIIA and GLUT4 (Fig. 3D). As shown previously, BAPTA-AM decreased the translocation of GLUT4 to the plasma membrane (Fig. 3D, panel i). BAPTA-AM also impaired MyoIIA translocation to the plasma membrane (Fig. 3D, panel j). Both GLUT4 and MyoIIA colocalized primarily in the cytoplasm in BAPTA-AM treated adipocytes (Fig. 3D panel l, arrows).

Next we wanted to determine whether increased levels of intracellular Ca^2+^ alone could induce MLCK phosphorylation as well as MyoIIA activity and recruitment to the plasma membrane in the absence of insulin stimulation. As expected, adipocytes treated with the calcium ionophore, A23187 alone did not stimulate glucose uptake (INS versus A23187, 5.4±0.61 versus 1.1±0.28, p<0.05) and also did not enhance glucose uptake in the presence of insulin (Fig. 3A). A23187 alone did induce phosphorylation of MLCK as well as the RLC associated with MyoIIA (Fig. 3B and C). Since MLCK activation is required for the translocation of MyoIIA we wanted to determine if elevated Ca^2+^ levels alone were sufficient to induce translocation. Upon treatment with A23187 (in the absence of insulin) MyoIIA translocated to the plasma membrane (Fig. 3D, panel n). A23187 was not sufficient to induce translocation of GLUT4 in the absence of insulin (Fig. 3D, panel m). These findings suggest that Ca^2+^ alone is able to stimulate the phosphorylation of MLCK which in turn phosphorylates and translocates MyoIIA to the plasma membrane (Fig. 3D, panel o and p, arrow indicating plasma membrane localization of MyoIIA only).

### ERK is required for MLCK phosphorylation during insulin stimulation

In order to determine whether there are other insulin-activated signaling pathways regulating MLCK phosphorylation, we wanted to examine known upstream regulators of MLCK. Both extracellular-signal regulated kinase (ERK) and calcium/calmodulin-dependent kinase II (CamKII) have been shown to be involved in insulin-stimulated glucose uptake as well as phosphorylation MLCK [3]–[6], [21]–[24]. To determine whether the insulin-induced phosphorylation of the MLCK was also due to either of these kinases we used both chemical inhibitors and targeted siRNA to assay for MLCK phosphorylation, glucose uptake and MyoIIA and GLUT4 localization.

**Figure 4:**
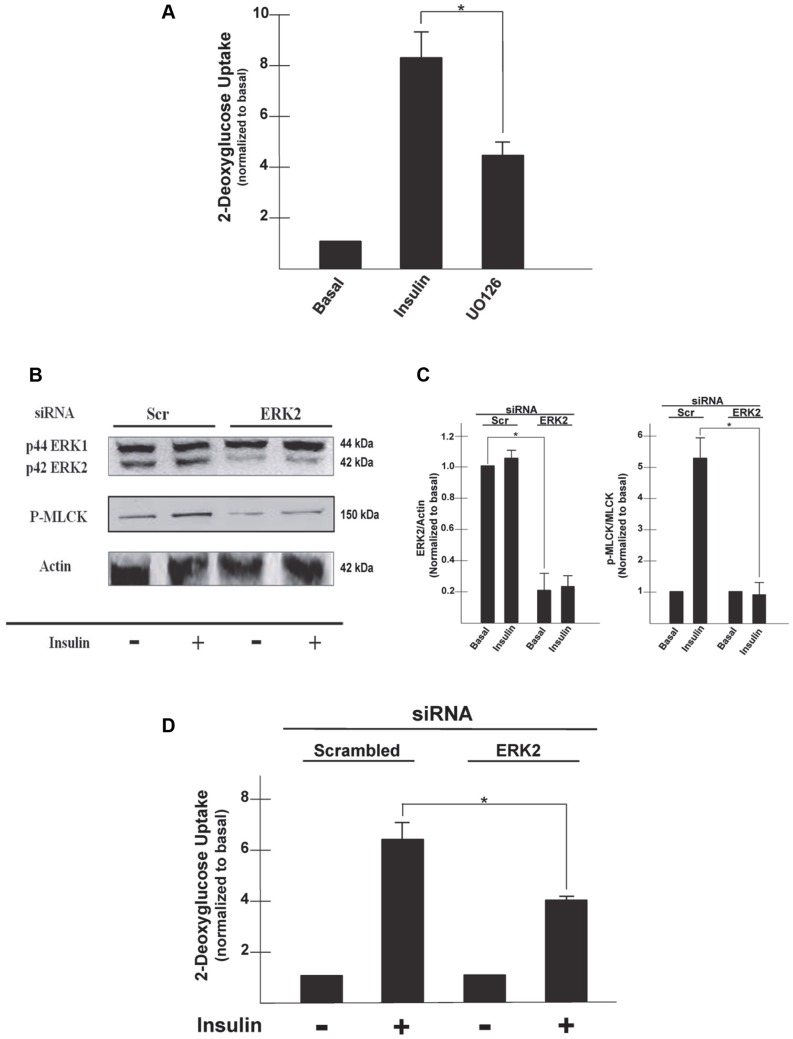
ERK is required for MLCK phosphorylation and insulin-stimulated glucose uptake. Serum starved 3T3-L1 adipocytes were pretreated with U0126 (25 µM) for 15 min and then either left untreated or stimulated with insulin (100 nM) for 20 min. (A) Untreated (Basal) or insulin stimulated (100 nM) adipocytes were incubated with [1-^14^C]-2-deoxy-D-glucose (0.1 µCi/well) and 5 mM glucose for an additional 10 min. Cells were then lysed and glucose uptake calculated as disintegrations per mg protein and expressed as percent of control. Results are the mean ± SEM of three independent experiments. *p<0.05. (B) Cells were electroporated with either siRNA for a scrambled sequence or ERK2, serum starved and then either left untreated or stimulated with insulin. Whole cell lysates were prepared, subjected to SDS-PAGE and then immunoblotted using antibodies against either an ERK antibody (that detects both p44 ERK1 and p42 ERK2), P-MLCK, or actin. (C) Determination of band densities was measured using ImageQuant software and are presented as either ERK2/actin or P-MLCK/MLCK and normalized to basal levels of Scr. Results are the mean ± SEM of three independent experiments. ERK2/actin levels were compared under basal conditions and P-MLCK/MLCK levels were compared after insulin stimulation in the Scr and ERK2 siRNA treated cells, *p<0.05. (D) Untreated (Basal) or insulin stimulated (100 nM) electroporated cells were then incubated in a solution containing [1-^14^C]-2-deoxy-D-glucose (0.1 µCi/well) and 5 mM glucose for an additional 10 min. Cells were lysed and glucose uptake calculated as disintegrations per mg protein. Results are mean ± SEM, *p<0.05.

Insulin stimulates ERK1/2 though activation of the RAS, Raf, MEK cascade. MEK is an immediate upstream activator of ERK1/2. The MEK inhibitor, U0126, not only decreases phosphorylation of ERK1/2 but also impairs insulin-stimulated glucose uptake in adipocytes [4]. ERK is also involved in the activation of MyoII. Previous studies have shown that in the presence of a constitutively active MEK there is an increase in phosphorylation of ERK1/2, MLCK, and RLC [35]. ERK1/2 has also been shown to directly phosphorylate MLCK initiating its activation [35]. To determine if ERK is required for the activation of MLCK during insulin-stimulated glucose uptake in adipocytes, we treated adipocytes with U0126 in the presence of insulin to examine the effect on glucose uptake and MyoIIA localization. In agreement with previous studies we show that U0126 impairs insulin-stimulated glucose uptake (Fig. 4A, Ins versus U0126, 8.3±0.98 versus 4.5±0.49, p<0.05) [4]. Inhibition of ERK1/2 also impaired both GLUT4 and MyoIIA from translocating to the plasma membrane upon insulin stimulation (Fig. 3D, panels q and r). Both GLUT4 and MyoIIA colocalized within the cytoplasm (Fig. 3D, panels s and t, arrow indicating colocalization in the cytoplasm). Since inhibition of ERK prevented translocation of MyoIIA, we wanted to determine both if ERK acts by regulating MLCK (inhibition of MLCK also impairs MyoIIA translocation) and which isoform of ERK was responsible for this regulation. In a previous study ERK2 alone was found to induce phosphorylation of MLCK and that in the absence of MLCK ERK2 was unable to phosphorylate RLC [35]. These findings suggest that ERK2 activates MLCK which then mediates the phosphorylation of RLC of MyoIIA. To examine the role of ERK2 in MLCK phosphorylation, we electroporated adipocytes with siRNA targeting ERK2 and then assayed for ERK2 expression, MLCK phosphorylation and glucose uptake. As shown by immunoblot analysis ERK2 levels were markedly reduced following siRNA treatment in comparison to ERK2 protein levels in cells electroporated with a scramble siRNA (Fig. 4B and C). Reduction in ERK2 proteins levels had no effect on the protein levels of ERK1 (Fig. 4B). In addition, our results show that insulin-stimulated phosphorylation of MLCK was markedly reduced in cells electroporated with ERK2 siRNA compared to that of cells electroporated with a scramble sequence (Fig. 4B and C). This suggests that ERK2 is an upstream activator of MLCK. Next, we wanted to determine the impact the reduced level of ERK2 would have on insulin-stimulated glucose uptake in the adipocytes. Figure 4D revealed no noticeable difference in the basal levels of glucose uptake into the cells with reduced levels of ERK2 compared to the control. However, under insulin-stimulated conditions, ERK2 deficient cells had approximately a 30% decrease in glucose uptake compared to cells electroporated with the scramble sequence (Fig, 4D, 6.4±0.66 versus 4.1±0.11, p<0.05). These results suggest a role for ERK2 in the regulation of MLCK and thus MyoIIA during insulin-stimulated glucose uptake in the adipocyte.

### CaMKII is required for MLCK phosphorylation during insulin stimulation

**Figure 5:**
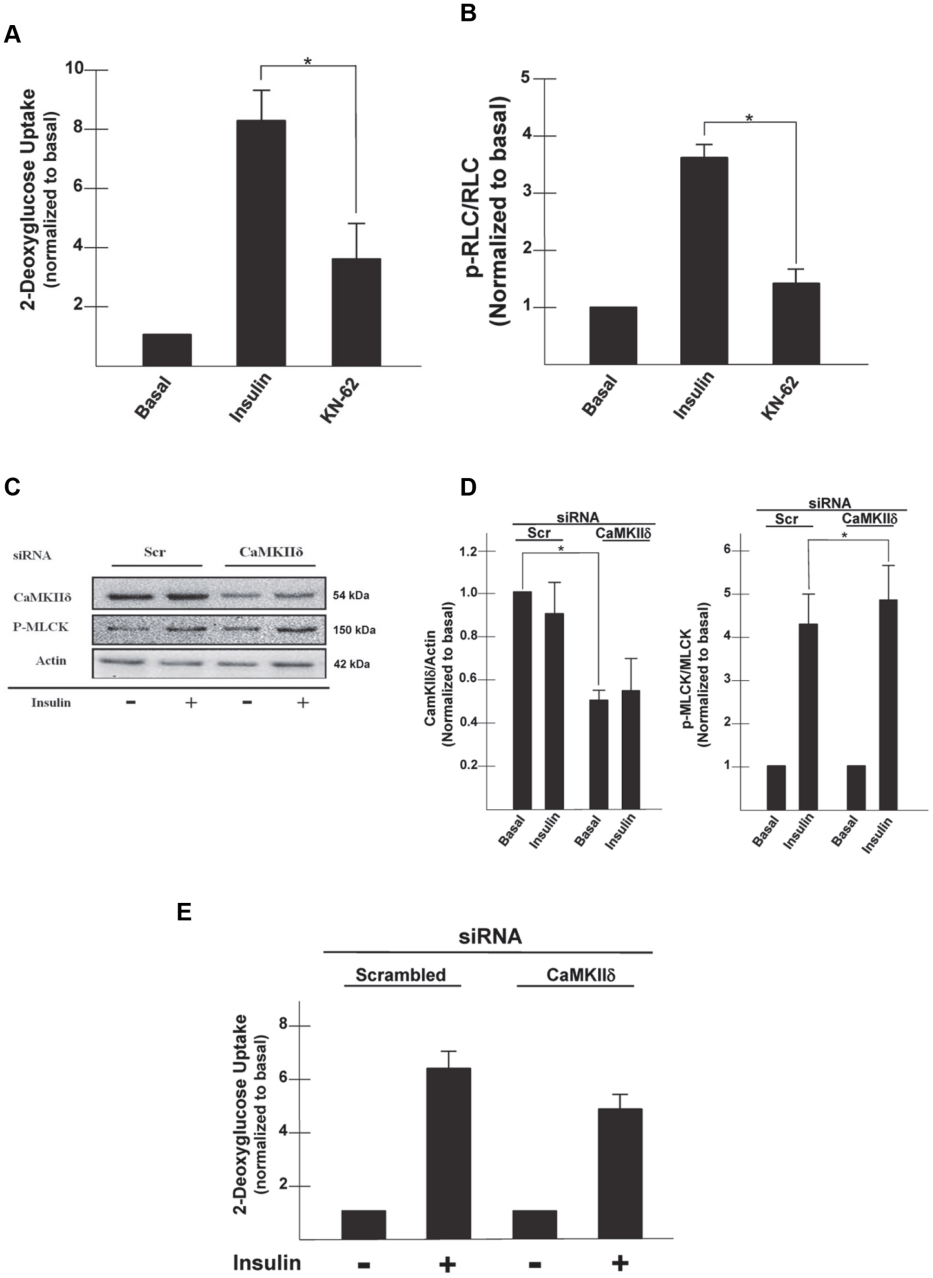
CaMKII but not CaMKIIδ is required for MLCK phosphorylation and glucose uptake in insulin-stimulated cells. 3T3-L1 adipocytes were serum starved for 4 h and the pretreated with KN-62 (100 µM) for 20 min and then either left untreated or stimulated with insulin (100 nM) for 20 min. (A) Untreated (Basal) or insulin stimulated (100 nM) adipocytes were incubated with [1-^14^C]-2-deoxy-D-glucose (0.1 µCi/well) and 5 mM glucose for an additional 10 min in the presence or absence of KN-62. Cells were then lysed and glucose uptake calculated as disintegrations per mg protein and expressed as percent of control. Results are the mean ± SEM of three independent experiments. *p<0.05. (B) MyoIIA was immunoprecipitated from cell lysates, analyzed by SDS-PAGE and then immunoblotted with anti-p-RLC. Results were quantified using NIH Image J and are presented as p-RLC/RLC and normalized to basal levels. Results are the mean ± SEM of three independent experiments. *p<0.05. 3T3-L1 adipocytes were electroporated with siRNA (1 nmole) for either CaMKIIδ or a scrambled sequence (Scr). After 72 h adipocytes were serum starved for 4 h and then either left untreated or stimulated with insulin (100 nM) for 20 min. (C) Whole cell lysates were prepared, subjected to SDS-PAGE and then immunoblotted using antibodies against CaMKIIδ, P-MLCK, or actin. (D) Determination of band densities was measured using ImageQuant software and are presented as either CaMKIIδ/actin or P-MLCK/MLCK and normalized to basal levels. Results are the mean ± SEM of three independent experiments. CaMKIIδ/actin levels were compared under basal conditions and P-MLCK/MLCK levels were compared after insulin stimulation in the Scr and CaMKIIδ siRNA treated cells, *p<0.05. (E) Untreated (Basal) or insulin stimulated (100 nM) electroporated cells were incubated in a solution containing [1-^14^C]-2-deoxy-D-glucose (0.1 µCi/well) and 5 mM glucose for an additional 10 min. Cells were lysed and glucose uptake calculated as disintegrations per mg protein. Results are the mean ±SEM of three independent experiments.

Inhibitor studies, using the CaMKII inhibitor, KN62, have implicated a role for CaMKII in insulin-stimulated glucose transport [36], [37]. CaMKII has multiples substrates, and it has been suggested to play a role in the regulation of both MLCK and ERK [38], [39]. What is not known is whether insulin stimulates MLCK and MyoIIA phosphorylation via the CaMKII pathway. To determine if CaMKII plays a role in the activation of MyoIIA, we treated adipocytes with the CaMKII inhibitor, KN-62 in the presence of insulin. Our studies show that KN-62 decreased insulin-stimulated glucose uptake by approximately 50% when compared to untreated cells (Fig. 5A, 8.3±0.97 versus 3.7±1.14, p<0.05). We next examined if inhibition of CaMKII impaired phosphorylation of the RLC of MyoIIA. Adipocytes treated with KN-62 in the presence of insulin had a 60% decrease in the levels of p-RLC associated with MyoIIA compared to levels in untreated cells (Fig. 5B, 3.6±0.22 versus 1.4±0.23, p<0.05).

A previous study examining the unconventional myosin, myosin IC (MyoIC) in adipocytes found that CaMKIIδ was able to phosphorylate MyoIC in vitro and subsequently activate its ATPase activity [37]. Thus we wanted to determine if CaMKIIδ was the CaMKII isoform regulating MLCK, and thus MyoIIA during insulin-stimulated glucose uptake. 3T3-L1 adipocytes were electroporated with siRNA targeting CaMKIIδ. After 72 h, cell lysates were collected and subjected to SDS-PAGE and immunoblot analysis. Our results show that there was a reduced level of CaMKIIδ expression in adipocytes electroporated with CaMKIIδ siRNA compared to those with the scramble sequence (Fig. 5C and D). We also observed that MLCK phosphorylation was not inhibited upon insulin stimulation in cells with CaMKIIδ siRNA and the levels were similar to those in cells treated with a scramble siRNA (Fig. 5C and D). To investigate if CaMKIIδ plays a role in insulin-stimulated glucose uptake, we assayed cells electroporated with a scramble sequence as well as cells electroporated with CaMKIIδ siRNA. As seen in Fig. 5E reduced levels of CaMKIIδ had no effect on glucose uptake under basal or insulin-stimulated conditions which suggests that while CaMKII is involved in MLCK-mediated glucose uptake, the CaMKIIδ isoform is not involved in insulin-stimulated glucose uptake in 3T3-L1 adipocytes.

## Discussion

Previous studies have demonstrated a role for members of the myosin family in GLUT4 trafficking [15]–[17], [40]–[44]. Myosin family members have been shown to traffic GLUT4 vesicles and to interact with components of the membrane docking and fusion machinery [16], [17], [41], [44]. Previous studies have shown a critical role for MyoII in GLUT4-mediated glucose uptake in adipocytes REF [14]–[17]. Since adipocytes express both MyoIIA and IIB, we wanted to determine the role of each in insulin-stimulated glucose uptake. While previous studies have used chemical inhibition, these inhibitors do not allow for the distinction between the two isoforms [16]. Although, more recently, the role of MyoIIA in insulin-stimulated glucose uptake was examined, it did not determine if MyoIIB also contributes to glucose uptake [17]. To better understand the role of the different MyoII isoforms we knocked down expression of both using siRNA. Our results reveal that only inhibition of MyoIIA through the use of an isoform-specific siRNA resulted in impaired insulin-stimulated glucose uptake. We were unable to knockdown MyoIIB without decreasing the expression of MyoIIA also. However since inhibition of both MyoIIA and IIB did not result in any further decrease in insulin-stimulated glucose when compared to inhibition of MyoIIA alone, then these findings suggest that only MyoIIA is involved in insulin-stimulated glucose uptake.

Previous studies have shown that MyoIIA is regulated by the phosphorylation of its RLC by MLCK [15], [16]. While our studies show that insulin signaling stimulates the phosphorylation of MLCK on Ser1760, a site that has been shown to increase its activity, other studies suggest that insulin is not phosphorylating MLCK via the PI3K pathway [32]–[34]. To determine the pathway(s) responsible for phosphorylating MLCK during insulin-stimulated glucose uptake, we examined pathways that were involved in both MLCK phosphorylation and insulin signaling. Three such signaling pathways that may function independently or in combination are calcium signaling and the MAPK and CaMKII pathways. Insulin is known to regulate calcium levels as well as the MAPK and CaMKII pathways, both of which are required for GLUT4-mediated glucose uptake [4]–[6]. Our studies demonstrate that calcium regulates MLCK and MyoIIA phosphorylation, MyoIIA translocation to the plasma membrane and insulin stimulated glucose uptake. Our studies are the first to show that BAPTA-AM (a calcium chelator) impaired MLCK phosphorylation and that calcium alone was able to phosphorylate MLCK in the absence of insulin (Fig. 3). These studies also demonstrated that calcium alone was sufficient to induce the translocation of MyoIIA to the membrane in the absence of insulin.

Insulin also activates multiple signaling pathways to facilitate GLUT4 trafficking and insulin-stimulated glucose uptake. The MAPK pathway has also been linked to insulin-stimulated glucose uptake but the downstream targets have not been identified [4]. Both ERK1 and 2 are able to directly phosphorylate MLCK which leads to increased phosphorylation of the RLC of MyoII [35]. Previous studies linking an inhibition of ERK2 to reduced levels of insulin-stimulated glucose uptake and GLUT4 translocation in muscle cells led us to examine the role of ERK2 in the phosphorylation of MLCK [45]. Our results reveal that chemical inhibition of MEK impaired insulin-stimulated glucose uptake by 50% in comparison to control levels. We were also able to show that ERK2 knockdown resulted in a decrease in insulin-stimulated phosphorylation of MLCK and also impaired insulin-stimulated glucose uptake. The reduced level of ERK2 resulted in a decrease in MLCK phosphorylation. Furthermore, confocal microscopy studies demonstrated that inhibition of ERK impaired MyoIIA translocation to the plasma membrane upon insulin stimulation.

Another signaling pathway regulating insulin-stimulated glucose uptake is the CaMKII pathway [39], [46]. CaMKII is a Ca^2+^-dependent kinase with multiple substrates which include ERK2 and MLCK [38], [39]. CaMKII has four isoforms with most cells expressing at least one (38). In this study the CaMKIIδ isoform was targeted for a possible role in insulin-stimulated glucose uptake due to its expression in adipocytes and its ability to phosphorylate MyoIC in vitro and subsequently activates its ATPase activity [43]. We investigated the possibility that CaMKIIδ could phosphorylate MLCK and thus the RLC of MyoIIA. Our results revealed that knockdown of the CaMKIIδ isoform did not inhibit insulin-stimulated glucose uptake in 3T3-L1 adipocytes or MLCK phosphorylation. One possible explanation of the discrepancy between these studies is that Toyoda et al. achieved a 90% reduction in CaMKIIδ expression and used a higher concentration of siRNA then that used in this study. It is possible that this greater reduction of CaMKIIδ protein levels was required to affect glucose uptake, while the modest reduction of CaMKIIδ expression in this study was not sufficient to inhibit MLCK phosphorylation or glucose uptake.

While our studies did not implicate CaMKIIδ in MyoIIA phosphorylation and glucose uptake, we did show that inhibition of CaMKII with chemical inhibitors did impair insulin-stimulated glucose uptake and phosphorylation of the RLC associated with MyoIIA. What is known is which CaMKII isoform is involved in MyoIIA regulation. Recent studies have implicated a role for CaMKII in ERK activation [24]. Studies using endothelial cells expressing a constitutively active CaMKII resulted in a significant increase in ERK activity (38, 45). Phosphorylation of ERK in these cells was inhibited by the CaMKII inhibitor, KN-62, and the MEK inhibitor U-0126 (38, 45). Furthermore ERK phosphorylation was inhibited in the absence of activated CaMKII (38, 45). Other studies using CaMKII inhibitors have shown a decrease in phosphorylation of ERK in smooth muscle cells and endothelial cells (39, 45). However, in a recent paper that showed chemical inhibition of CaMKII resulted in a decrease in phosphorylated ERK; they were not able to find a subsequent reduction in RLC phosphorylation (45). Our studies implicate a possible scenario in which insulin signaling increases intracellular Ca^2+^ levels which activates a CamKII-ERK2-MLCK-RLC/MyoIIA cascade to regulate insertion of GLUT4 vesicles at the plasma membrane during insulin-stimulated glucose uptake in adipocytes. These results are similar to those reported in a recent study showing that translocation of Aquaporin-1 in renal proximal tubules also involved an ERK-MLCK-RLC/MyoII cascade [47]. However, these studies did not implicate calcium or CaMKII in the activation of this signaling cascade.

In summary, our studies show that MLCK activation requires both, an increase in intracellular Ca^+2^ and activation of ERK to phosphorylate and translocate MyoIIA to the plasma membrane to facilitate insulin-stimulated glucose uptake. We also show that while these effects are mediated by insulin, Ca^+2^ alone is sufficient to phosphorylate MLCK and thus MyoIIA. Our results also demonstrate that while CaMKIIδ regulates other myosin family members, it does not stimulate phosphorylation of either MLCK or MyoIIA upon insulin stimulation. Collectively, our studies have elucidated the insulin-induced signaling components required for MLCK and MyoIIA activation necessary for GLUT4-mediated glucose uptake. Our findings further elucidate the role of cytoskeletal components involved in insulin-stimulated glucose uptake and provide further insight on the cellular mechanisms regulating GLUT4 trafficking.

## References

[1] Cushman SW, Wardzala LJ (1980) Potential mechanism of insulin action on glucose transport in the isolated rat adipose cell. Apparent translocation of intracellular transport systems to the plasma membrane. J Biol Chem 255: 4758–4762. 6989818

[2] Stockli J, Fazakerley DJ, James DE (2011) GLUT4 exocytosis. J Cell Sci 124: 4147–4159. 22247191 10.1242/jcs.097063 PMC3258103

[3] Saltiel AR, Pessin JE (2002) Insulin signaling pathways in time and space. Trends Cell Biol 12: 65–71. 11849969 10.1016/s0962-8924(01)02207-3

[4] Harmon AW, Paul DS, Patel YM (2004) MEK inhibitors impair insulin-stimulated glucose uptake in 3T3-L1 adipocytes. Am J Physiol Endocrinol Metab 287: E758–766. 15172888 10.1152/ajpendo.00581.2003

[5] Whitehead JP, Molero JC, Clark S, Martin S, Meneilly G, et al (2001) The role of Ca2+ in insulin-stimulated glucose transport in 3T3-L1 cells. J Biol Chem 276: 27816–27824. 11375387 10.1074/jbc.M011590200

[6] Worrall DS, Olefsky JM (2002) The effects of intracellular calcium depletion on insulin signaling in 3T3-L1 adipocytes. Mol Endocrinol 16: 378–389. 11818508 10.1210/mend.16.2.0776

[7] Pang ZP, Sudhof TC (2010) Cell biology of Ca2+-triggered exocytosis. Curr Opin Cell Biol 22: 496–505. 20561775 10.1016/j.ceb.2010.05.001 PMC2963628

[8] Yu H, Rathore SS, Davis EM, Ouyang Y, Shen J (2013) Doc2b promotes GLUT4 exocytosis by activating the SNARE-mediated fusion reaction in a calcium- and membrane bending-dependent manner. Mol Biol Cell 24: 1176–1184. 23427263 10.1091/mbc.E12-11-0810 PMC3623638

[9] Kanzaki M, Pessin JE (2001) Insulin-stimulated GLUT4 translocation in adipocytes is dependent upon cortical actin remodeling. J Biol Chem 276: 42436–42444. 11546823 10.1074/jbc.M108297200

[10] Omata W, Shibata H, Li L, Takata K, Kojima I (2000) Actin filaments play a critical role in insulin-induced exocytotic recruitment but not in endocytosis of GLUT4 in isolated rat adipocytes. Biochem J 346 Pt 2: 321–328. PMC1220856 10677349

[11] Bresnick AR (1999) Molecular mechanisms of nonmuscle myosin-II regulation. Curr Opin Cell Biol 11: 26–33. 10047526 10.1016/s0955-0674(99)80004-0

[12] Heissler SM, Manstein DJ (2013) Nonmuscle myosin-2: mix and match. Cell Mol Life Sci 70: 1–21. 22565821 10.1007/s00018-012-1002-9 PMC3535348

[13] Neco P, Giner D, Viniegra S, Borges R, Villarroel A, et al (2004) New roles of myosin II during vesicle transport and fusion in chromaffin cells. J Biol Chem 279: 27450–27457. 15069078 10.1074/jbc.M311462200

[14] Steimle PA, Fulcher FK, Patel YM (2005) A novel role for myosin II in insulin-stimulated glucose uptake in 3T3-L1 adipocytes. Biochem Biophys Res Commun 331: 1560–1565. 15883051 10.1016/j.bbrc.2005.04.082

[15] Choi YO, Ryu HJ, Kim HR, Song YS, Kim C, et al (2006) Implication of phosphorylation of the myosin II regulatory light chain in insulin-stimulated GLUT4 translocation in 3T3-F442A adipocytes. Exp Mol Med 38: 180–189. 16672772 10.1038/emm.2006.22

[16] Fulcher FK, Smith BT, Russ M, Patel YM (2008) Dual role for myosin II in GLUT4-mediated glucose uptake in 3T3-L1 adipocytes. Exp Cell Res 314: 3264–3274. 18773891 10.1016/j.yexcr.2008.08.007 PMC2626409

[17] Chung le TK, Hosaka T, Harada N, Jambaldorj B, Fukunaga K, et al (2010) Myosin IIA participates in docking of Glut4 storage vesicles with the plasma membrane in 3T3-L1 adipocytes. Biochem Biophys Res Commun 391: 995–999. 19968963 10.1016/j.bbrc.2009.12.004

[18] Hartshorne DJ (1998) Myosin phosphatase: subunits and interactions. Acta Physiol Scand 164: 483–493. 9887971 10.1046/j.1365-201X.1998.00447.x

[19] Kamm KE, Stull JT (2001) Dedicated myosin light chain kinases with diverse cellular functions. J Biol Chem 276: 4527–4530. 11096123 10.1074/jbc.R000028200

[20] Xia D, Stull JT, Kamm KE (2005) Myosin phosphatase targeting subunit 1 affects cell migration by regulating myosin phosphorylation and actin assembly. Exp Cell Res 304: 506–517. 15748895 10.1016/j.yexcr.2004.11.025

[21] Hong F, Haldeman BD, Jackson D, Carter M, Baker JE, et al (2011) Biochemistry of smooth muscle myosin light chain kinase. Arch Biochem Biophys 510: 135–146. 21565153 10.1016/j.abb.2011.04.018 PMC3382066

[22] Nguyen DH, Catling AD, Webb DJ, Sankovic M, Walker LA, et al (1999) Myosin light chain kinase functions downstream of Ras/ERK to promote migration of urokinase-type plasminogen activator-stimulated cells in an integrin-selective manner. J Cell Biol 146: 149–164. 10402467 10.1083/jcb.146.1.149 PMC2199739

[23] Bessard A, Coutant A, Rescan C, Ezan F, Fremin C, et al (2006) An MLCK-dependent window in late G1 controls S phase entry of proliferating rodent hepatocytes via ERK-p70S6K pathway. Hepatology 44: 152–163. 16799973 10.1002/hep.21222

[24] Tansey MG, Luby-Phelps K, Kamm KE, Stull JT (1994) Ca(2+)-dependent phosphorylation of myosin light chain kinase decreases the Ca2+ sensitivity of light chain phosphorylation within smooth muscle cells. J Biol Chem 269: 9912–9920. 8144585

[25] Green H, Meuth M (1974) An established pre-adipose cell line and its differentiation in culture. Cell 3: 127–133. 4426090 10.1016/0092-8674(74)90116-0

[26] Student AK, Hsu RY, Lane MD (1980) Induction of fatty acid synthetase synthesis in differentiating 3T3-L1 preadipocytes. J Biol Chem 255: 4745–4750. 7372608

[27] Mitra P, Zheng X, Czech MP (2004) RNAi-based analysis of CAP, Cbl, and CrkII function in the regulation of GLUT4 by insulin. J Biol Chem 279: 37431–37435. 15258163 10.1074/jbc.C400180200

[28] Welsh GI, Leney SE, Lloyd-Lewis B, Wherlock M, Lindsay AJ, et al (2007) Rip11 is a Rab11- and AS160-RabGAP-binding protein required for insulin-stimulated glucose uptake in adipocytes. J Cell Sci 120: 4197–4208. 18003705 10.1242/jcs.007310

[29] Williams D, Hicks SW, Machamer CE, Pessin JE (2006) Golgin-160 is required for the Golgi membrane sorting of the insulin-responsive glucose transporter GLUT4 in adipocytes. Mol Biol Cell 17: 5346–5355. 17050738 10.1091/mbc.E06-05-0386 PMC1679696

[30] Laemmli UK (1970) Cleavage of structural proteins during the assembly of the head of bacteriophage T4. Nature 227: 680–685. 5432063 10.1038/227680a0

[31] Paul DS, Harmon AW, Winston CP, Patel YM (2003) Calpain facilitates GLUT4 vesicle translocation during insulin-stimulated glucose uptake in adipocytes. Biochem J 376: 625–632. 12974673 10.1042/BJ20030681 PMC1223814

[32] Conti MA, Adelstein RS (1981) The relationship between calmodulin binding and phosphorylation of smooth muscle myosin kinase by the catalytic subunit of 3′:5′ cAMP-dependent protein kinase. J Biol Chem 256: 3178–3181. 6259152

[33] Ikebe M, Reardon S (1990) Phosphorylation of smooth myosin light chain kinase by smooth muscle Ca2+/calmodulin-dependent multifunctional protein kinase. J Biol Chem 265: 8975–8978. 2160950

[34] Goeckeler ZM, Masaracchia RA, Zeng Q, Chew TL, Gallagher P, et al (2000) Phosphorylation of myosin light chain kinase by p21-activated kinase PAK2. J Biol Chem 275: 18366–18374. 10748018 10.1074/jbc.M001339200

[35] Klemke RL, Cai S, Giannini AL, Gallagher PJ, de Lanerolle P, et al (1997) Regulation of cell motility by mitogen-activated protein kinase. J Cell Biol 137: 481–492. 9128257 10.1083/jcb.137.2.481 PMC2139771

[36] Brozinick JT Jr, Reynolds TH, Dean D, Cartee G, Cushman SW (1999) 1-[N, O-bis-(5-isoquinolinesulphonyl)-N-methyl-L-tyrosyl]-4- phenylpiperazine (KN-62), an inhibitor of calcium-dependent camodulin protein kinase II, inhibits both insulin- and hypoxia-stimulated glucose transport in skeletal muscle. Biochem J 339 (Pt 3): 533–540. PMC1220187 10215590

[37] Yip MF, Ramm G, Larance M, Hoehn KL, Wagner MC, et al (2008) CaMKII-mediated phosphorylation of the myosin motor Myo1c is required for insulin-stimulated GLUT4 translocation in adipocytes. Cell Metab 8: 384–398. 19046570 10.1016/j.cmet.2008.09.011

[38] Soderling TR, Stull JT (2001) Structure and regulation of calcium/calmodulin-dependent protein kinases. Chem Rev 101: 2341–2352. 11749376 10.1021/cr0002386

[39] Illario M, Monaco S, Cavallo AL, Esposito I, Formisano P, et al (2009) Calcium-calmodulin-dependent kinase II (CaMKII) mediates insulin-stimulated proliferation and glucose uptake. Cell Signal 21: 786–792. 19171190 10.1016/j.cellsig.2009.01.022

[40] Bose A, Guilherme A, Robida SI, Nicoloro SM, Zhou QL, et al (2002) Glucose transporter recycling in response to insulin is facilitated by myosin Myo1c. Nature 420: 821–824. 12490950 10.1038/nature01246

[41] Bose A, Robida S, Furcinitti PS, Chawla A, Fogarty K, et al (2004) Unconventional myosin Myo1c promotes membrane fusion in a regulated exocytic pathway. Mol Cell Biol 24: 5447–5458. 15169906 10.1128/MCB.24.12.5447-5458.2004 PMC419880

[42] Yoshizaki T, Imamura T, Babendure JL, Lu JC, Sonoda N, et al (2007) Myosin 5a is an insulin-stimulated Akt2 (protein kinase Bbeta) substrate modulating GLUT4 vesicle translocation. Mol Cell Biol 27: 5172–5183. 17515613 10.1128/MCB.02298-06 PMC1951956

[43] Toyoda T, An D, Witczak CA, Koh HJ, Hirshman MF, et al (2011) Myo1c regulates glucose uptake in mouse skeletal muscle. J Biol Chem 286: 4133–4140. 21127070 10.1074/jbc.M110.174938 PMC3039380

[44] Boguslavsky S, Chiu T, Foley KP, Osorio-Fuentealba C, Antonescu CN, et al (2012) Myo1c binding to submembrane actin mediates insulin-induced tethering of GLUT4 vesicles. Mol Biol Cell 23: 4065–4078. 22918957 10.1091/mbc.E12-04-0263 PMC3469521

[45] Berti L, Gammeltoft S (1999) Leptin stimulates glucose uptake in C2C12 muscle cells by activation of ERK2. Mol Cell Endocrinol 157: 121–130. 10619403 10.1016/s0303-7207(99)00154-9

[46] Konstantopoulos N, Marcuccio S, Kyi S, Stoichevska V, Castelli LA, et al (2007) A purine analog kinase inhibitor, calcium/calmodulin-dependent protein kinase II inhibitor 59, reveals a role for calcium/calmodulin-dependent protein kinase II in insulin-stimulated glucose transport. Endocrinology 148: 374–385. 17008397 10.1210/en.2006-0446

[47] Zhang J, An Y, Gao J, Han J, Pan X, et al (2012) Aquaporin-1 translocation and degradation mediates the water transportation mechanism of acetazolamide. PLoS One 7: e45976. 23029347 10.1371/journal.pone.0045976 PMC3448731

